# Median Arcuate Ligament Syndrome: A Case Report on a Rare Disease and Variant Hepatic Arterial Anatomy

**DOI:** 10.7759/cureus.64514

**Published:** 2024-07-14

**Authors:** Dillon Rogando, Dhruv Patel, Jeffrey Robles, Tara Ranjbar, Sourodip Mukharjee, Debra H Chan, Erika Clarke, Indraneil Mukherjee

**Affiliations:** 1 General Surgery, City College of New York School of Medicine, New York, USA; 2 Surgery, City College of New York School of Medicine, New York, USA; 3 General Surgery, Staten Island University Hospital, New York, USA; 4 Minimally Invasive Surgery, Staten Island University Hospital, New York, USA

**Keywords:** dunbar syndrome, median arcuate ligament syndrome, celiac axis syndrome, the median arcuate ligament compresses the celiac trunk, atypical mals

## Abstract

Median arcuate ligament syndrome (MALS) is a rare gastroenterological illness that arises from the compression of the celiac trunk by the fibrous arch known as the median arcuate ligament, which connects the muscular tendon of the diaphragm to the vertebral column. It is hypothesized that this syndrome arises due to the inadequate caudal migration of the celiac trunk during embryogenesis, although the exact pathophysiology behind this disease process remains unclear. While MALS is classically associated with a triad of post-prandial pain, weight loss, and epigastric bruit, the triad is often incomplete due to variations in vascular structures with collateral circulation from adjacent vessels. When symptoms are present, they can be vague and often characterized as unexplained nausea, vomiting, diarrhea, or flatulence. Frequently, MALS is identified incidentally upon imaging of the abdomen in response to these nonspecific complaints. We present the case of a patient suffering from MALS in which a rare anatomic variant of the celiac trunk was identified.

## Introduction

The median arcuate ligament is a fibrous arch formed from the coalescence of the diaphragmatic crura, the latter anchoring the tendinous attachment of the diaphragm to the lumbar vertebral bodies. In rare instances, the celiac trunk, arising from the abdominal aorta at the level of T12/L1, may undergo mechanical compression by this fibrous arch [1]. Disruption in arterial perfusion to structures supplied by the celiac trunk manifests in symptoms such as vague postprandial abdominal pain, weight loss, nausea, and vomiting [2]. Together, this clinical syndrome is commonly referred to as median arcuate ligament syndrome also known as MALS. The incidence of MALS is low; it occurs in approximately 2/100,000 individuals [1]. It may be largely underreported due to the nonspecific and intermitted nature of associated symptoms. Up to 87% of patients are asymptomatic and are incidentally identified on computed tomography (CT) [3].

When MALS is suspected, imaging modalities including Doppler ultrasound of the celiac trunk, magnetic resonance angiography, and CT angiography (CTA) are routinely used. The current gold-standard test for diagnosing MALS is three-dimensional CTA. Using this imaging technique, clinicians can determine the degree of artery compression and identify potential collateral vessels and anatomic anomalies [4]. We report the case of an anatomic anomaly involving the right hepatic artery, an artery normally arising as a tributary of the common hepatic artery and celiac trunk.

## Case presentation

**Figure 1:**
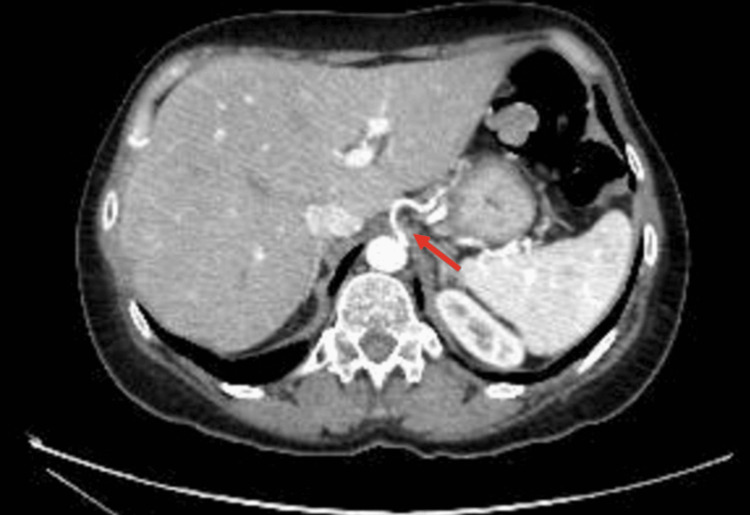
Axial CT demonstrating stenosis of the left gastric artery as it arises from the aorta The red arrow denotes the left gastric artery, slightly narrow at its origin, from the abdominal aorta

**Figure 2:**
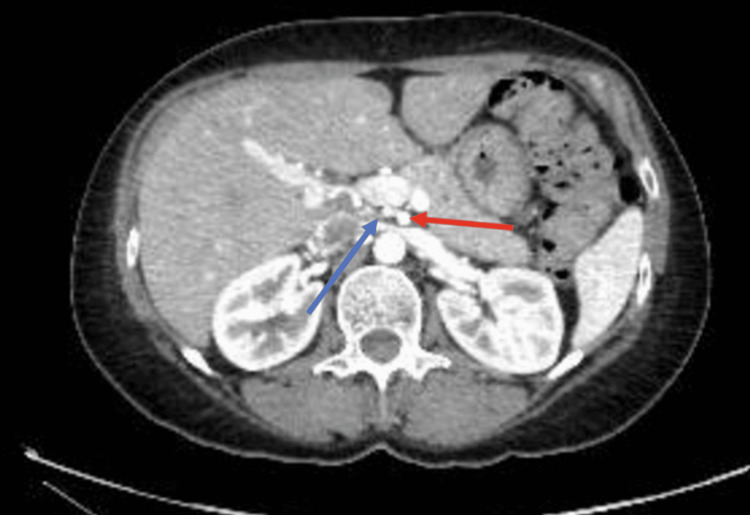
Axial CT demonstrating the right hepatic artery as it arises from the superior mesenteric artery The blue arrow denotes the proximal portion of the right hepatic artery. The red arrow denotes the superior mesenteric artery

**Figure 3:**
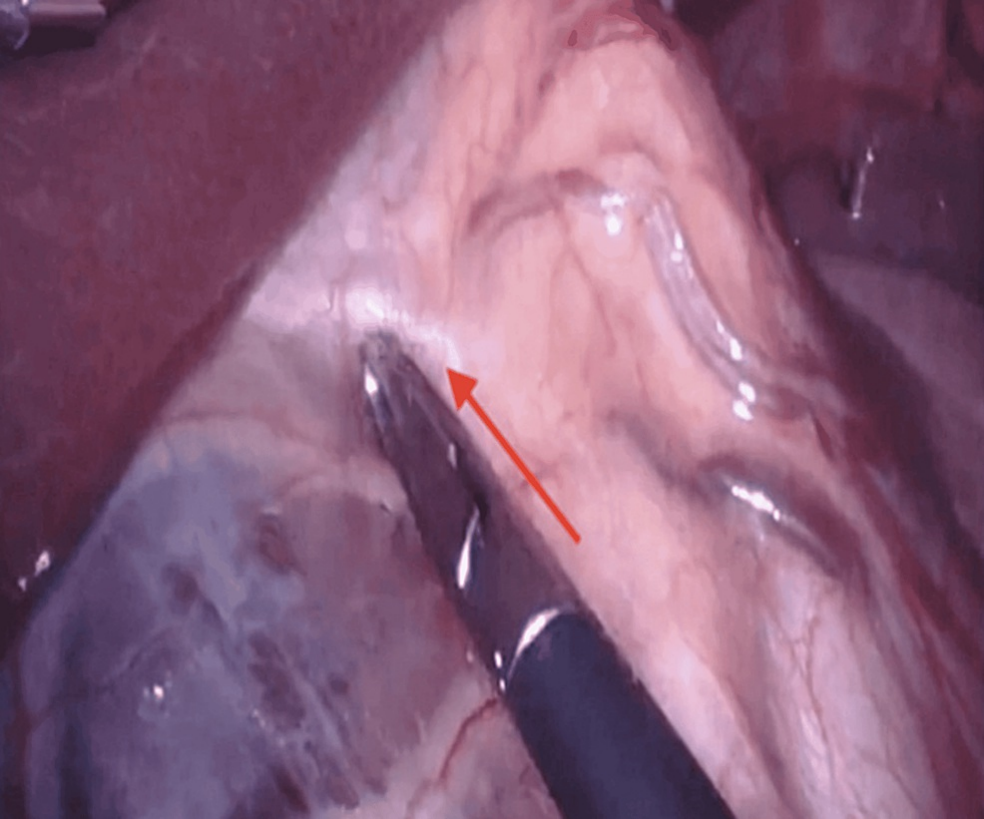
Intraoperative view of an accessory left hepatic artery arising from the left gastric artery at its origin The red arrow denotes an accessory left hepatic artery

We report the case of a 49-year-old female with a past medical history significant for coronary artery disease, dyslipidemia, and bilateral lower extremity venous insufficiency with a 50-pack-year smoking history, who presented with recurrent postprandial abdominal pain for the past 13 years. She described the pain as diffusely dull and crampy. Along with abdominal pain, the patient endorsed concurrent nausea with oral food intake and associated flushing. She was evaluated by several physicians who had performed upper gastrointestinal series, gastric emptying studies, breath testing for small intestinal bowel overgrowth, and an esophagogastroduodenoscopy (EGD) which were unremarkable. An EGD performed one year before presenting in the office demonstrated mild gastritis. After initial consultation in the office, the patient underwent CTA which showed narrowing of the celiac trunk and associated left gastric artery, indicative of MALS although no explicit compression by the median arcuate ligament was evident on CT (Figure 1). The right hepatic artery was also seen arising from the superior mesenteric artery also known as the SMA (Figure 2). Due to the symptomatic presentation, in combination with prior imaging studies, the patient underwent robotic resection of the median arcuate ligament and transversus abdominis plane block for abdominal pain secondary to celiac trunk compression syndrome (Video 1). Intraoperatively, a flexible liver retractor was placed in the left upper quadrant to provide lateral traction against the liver. The caudal segment of the hepato-gastric ligament, pars flaccida, was dissected, and an accessory left hepatic artery could be visualized arising from the origin of the left gastric artery. The patient was repositioned in reverse Trendelenburg, and dissection of the peritoneum commenced. The common hepatic artery was visualized, and the peritoneum between the common hepatic and left gastric artery was dissected. An enlarged lymph node adjacent to the common hepatic artery was identified, after which it was dissected and repositioned. The peritoneum surrounding the left gastric artery was reduced, and a blue vessel loop with clips was attached to avoid injury to the vascular structure and provide traction on the vessel. Extensive dissection of the peritoneum was performed, and the aberrant anatomy was visualized. The left hepatic artery was identified as it arose from the common hepatic artery and subsequent celiac trunk while an accessory left hepatic artery was identified as a branch off the left gastric artery (Figure 3). The right hepatic artery could be seen arising from the descending portion of the SMA, after which the crural fibers of the diaphragm were transected bilaterally with electrocautery using a combination book, bipolar, Maryland, and electrocautery scissors. The junction of the aorta and gastric artery was visualized in proximity to the median arcuate ligament, with the latter transected with a hook 3 cm cranially. Intraoperative indocyanine green fluorescence was deployed, confirming no compression of vascular structures by the median arcuate ligament during inspiration and expiration. No intraoperative bleeding was noted, and Vistaseal was injected at this site. The flexible liver retractor and other instrumentation were removed, and the skin was closed with 4-0 Monocryl, Benzoin, and Steri-Strips. The patient was successfully extubated and transferred to the recovery unit.

**Video 1:** Release of the median arcuate ligament

Postoperatively, the patient required 1 push of ephedrine for systolic blood pressure in the 70s, which subsequently improved to 97/50. Additionally, she reported generalized, non-radiating, and non-focal chest pain, which was controlled with IV Tylenol and oral non-steroidal anti-inflammatory drugs. Evaluation on postoperative day 15 demonstrated no postoperative complications or recurrence of symptoms. 

Written informed consent was obtained from the patient regarding the use of their medical history for educational purposes.

## Discussion

MALS, termed Dunbar syndrome, is an uncommon condition resulting from the mechanical compression of the celiac trunk by the median arcuate ligament of the diaphragm. It was first described by the physician Harjola in 1963, wherein a patient presented with postprandial epigastric pain and was later found, intraoperatively, to suffer from the compression of the celiac trunk by a thick fibrous band identified as the median arcuate ligament [5]. The incidence of MALS is estimated to be 2/100,000 in the general population, with a ratio of distribution among women and men of 3:1, respectively [6]. This gender disparity is not well understood; however, one mechanism that has been proposed is insufficient caudal migration of the celiac trunk during embryogenesis [7]. During early embryonic development, the celiac trunk and SMA form from paired segmental arteries that converge in the midline and migrate caudally during the formal development of the gut tube [7]. Faulty caudal migration results in a superiorly displaced celiac trunk which may be subsequently compressed by the median arcuate ligament above. Reportedly, the median arcuate ligament crosses the celiac trunk at the level of L1 in 10-24% of the population. This statistic may be underreported/underestimated, as a large percentage of individuals are asymptomatic, and the symptoms associated with MALS are highly variable. Symptoms ranging from crampy or diffuse abdominal pain and diarrhea to substernal chest pain have been reported as the initial presenting symptoms [8,9].

The current mainstay diagnostic tool used to visualize MALS is CT [10]. Utilization of CT allows for the direct visualization of the characteristic narrowing of the proximal celiac trunk as well as the post-stenotic dilatation caused by the aberrantly positioned median arcuate ligament. Other methods that have been used in the diagnosis of MALS include Doppler ultrasound and CTA. Doppler ultrasound in MALS can show increased peak systolic velocity at the celiac trunk, particularly during expiration and while in the supine position. CTA findings characteristic of MALS can show narrowing in the proximal celiac trunk, which commonly resembles a "hooked" appearance [11]. 

Laparoscopic median arcuate ligament release is the mainstay surgical treatment for MALS. However, studies are being performed to also assess the effectiveness of robot-assisted surgery, which is a new method recently being utilized to treat MALS. Research has shown that both procedures can be performed with minimal morbidity and mortality and that outcomes are equivalent [12]. Differences in the two processes lie in the length of completion and levels of postoperative chronic abdominal pain [12,13]. In this domain, a laparoscopic approach remains superior. As further research is conducted on methods to increase the efficiency of robot-assisted surgery, in combination with healthcare professionals becoming more familiar with this technology, robot-assisted surgery has the potential to become the future mainstay surgical treatment of MALS.

From what is known thus far, there is great anatomic variability in the origin of the celiac trunk. Certain variants, such as the right hepatic artery or accessory right hepatic artery arising from the SMA, occur frequently and have been observed in at least 15% of individuals [14]. In the anatomical variant (Hiatt IV classification), as seen in our patient in which an accessory left hepatic artery and right hepatic artery arising from the SMA was present, the reported incidence has been as low as 0.82% [14-16]. Upon review of the literature, this variant has been mainly reported postmortem and has not been reported in an individual with concomitant MALS. Careful preoperative assessment of anatomic variation is important for minimizing the risk of intraoperative vascular injury.

## Conclusions

Symptoms and clinical presentation of MALS are highly variable due to various anatomical variants among patients and the degree of compression imposed by the fibrous arch. While its exact cause remains elusive, potential mechanisms such as embryonic maldevelopment have been proposed. The diagnosis of MALS primarily relies on imaging modalities like CT, Doppler ultrasound, and CTA. When a diagnosis is made, laparoscopic median arcuate ligament release is the preferred method of surgical intervention. Furthermore, the variability in vascular anatomy underscores the importance of meticulous preoperative assessment to mitigate the risk of intraoperative complications. As our understanding of MALS and its management continues to evolve, interdisciplinary collaboration and technological advancements hold the key to improving patient care and outcomes in the future.
